# Targeted H3R26 Deimination Specifically Facilitates Estrogen Receptor Binding by Modifying Nucleosome Structure

**DOI:** 10.1371/journal.pgen.1004613

**Published:** 2014-09-11

**Authors:** Michael J. Guertin, Xuesen Zhang, Lynne Anguish, Sohyoung Kim, Lyuba Varticovski, John T. Lis, Gordon L. Hager, Scott A. Coonrod

**Affiliations:** 1 Lab of Receptor Biology and Gene Expression, National Cancer Institute, Bethesda, Maryland, United States of America; 2 State Key Laboratory of Reproductive Medicine, Nanjing Medical University, Nanjing, Jiangsu, China; 3 Baker Institute for Animal Health, College of Veterinary Medicine, Cornell University, Ithaca, New York, United States of America; 4 Department of Molecular Biology and Genetics, Cornell University, Ithaca, New York, United States of America; Cancer Research UK Cambridge Research Institute, United Kingdom

## Abstract

Transcription factor binding to DNA *in vivo* causes the recruitment of chromatin modifiers that can cause changes in chromatin structure, including the modification of histone tails. We previously showed that estrogen receptor (ER) target gene activation is facilitated by peptidylarginine deiminase 2 (PAD2)-catalyzed histone H3R26 deimination (H3R26Cit). Here we report that the genomic distributions of ER and H3R26Cit in breast cancer cells are strikingly coincident, linearly correlated, and observed as early as 2 minutes following estradiol treatment. The H3R26Cit profile is unlike that of previously described histone modifications and is characterized by sharp, narrow peaks. Paired-end MNase ChIP-seq indicates that the charge-neutral H3R26Cit modification facilitates ER binding to DNA by altering the fine structure of the nucleosome. Clinically, we find that PAD2 and H3R26Cit levels correlate with ER expression in breast tumors and that high PAD2 expression is associated with increased survival in ER+ breast cancer patients. These findings provide insight into how transcription factors gain access to nucleosomal DNA and implicate PAD2 as a novel therapeutic target for ER+ breast cancer.

## Author Summary

Transcription factors bind to DNA to activate and repress gene transcription. Many transcription factors, particularly nuclear receptors, associate with their cognate DNA element in a highly dynamic manner *in vivo*. Highly acetylated histone tails and DNase sensitive chromatin are amenable to the initial binding of transcription factors. Upon binding to DNA, transcription factor binding recruits remodelers and coactivators that can cause a concomitant increase in accessibility and acetylation. Herein, we show that estrogen receptor recruitment of a histone deiminase causes the positively charged H3R26 residue to be neutralized. This modification changes the fine structure of the nucleosome particle and facilitates estrogen receptor binding. Lastly, we find that high deiminase expression is associated with increased survival in estrogen receptor-positive breast cancer patients.

## Introduction

The nucleosome represents the fundamental unit of chromatin and consists of 147 bp of DNA wrapped ∼1.7 times around the histone octamer core particle [1]. The N-terminal tails of histones are disordered and reside outside of core nucleosome/DNA structure but they harbor amino acid residues that can be post-translationally modified to regulate many facets of transcription. These modifications can influence transcription factor access to nucleosomal DNA by modulating electrostatic interactions between histones and DNA. However, the means by which different transcription factors (TF) gain access to their DNA elements in the context of chromatin remain to be fully elucidated [2].

Recent studies of nuclear receptors have shown that androgen receptor (AR) [3], glucocorticoid receptor (GR) [4], [5], and progesterone receptor (PR) [6] access their respective DNA elements through extensive reorganization of nucleosomes using chromatin remodeling enzymes that cause concomitant increases in DNase accessibility. While estrogen receptor (ER) also interacts with nucleosome remodelers to maintain the accessible chromatin state [7]–[9], we find that concomitant increases in accessibility at ER binding sites are less prevalent than with other nuclear receptors. We have previously reported that PAD enzymes catalyze the conversion of protein arginine residues to neutrally charged citrulline in a process called deimination or, alternatively, citrullination [10], [11]. More specifically, we found that PAD2-mediated deimination of histone H3 arginine 26 (H3R26Cit) is important for estradiol (E2)-mediated activation of ER-target genes [12]. Here, we examine this E2-induced deamination at high temporal and spatial resolution and test the hypothesis that PAD2 facilitates ER/DNA binding by neutralizing the H3R26 charge of local nucleosomes via deimination, thereby weakening histone tail-DNA interactions and destabilizing DNA interactions with the core nucleosome particle.

## Results

### ER and H3R26Cit are highly concordant

**Figure 1:**
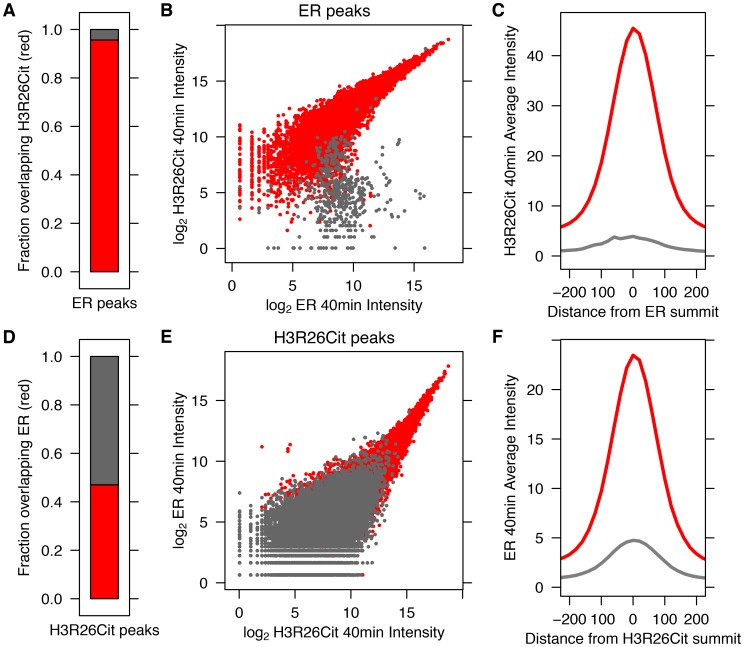
ER Binding and H3R26 citrullination are highly correlated after estrogen treatment. A) Ninety–five percent of ER peaks overlap H3R26Cit peaks (red bar). B) The H3R26Cit raw intensity is strongly correlated (Pearson coefficient of 0.92) with ER intensity at ER peaks that overlap H3R26Cit peaks (red points); grey points are not enriched for H3R26Cit. C) The composite H3R26Cit signal is centered on the ER summit at ER binding sites that overlap H3R26Cit peaks (red trace). In contrast, the grey trace is composite H3R26Cit signal at the 5% of ER peaks that do not overlap H3R26Cit peaks. D) Forty-seven percent of H3R26Cit peaks overlap ER peaks. E) The ER raw intensity is linearly correlated (Pearson coefficient = 0.94) with H3R26Cit intensity at all H3R26Cit sites. F) The composite ER signal at H3R26Cit binding sites that overlap H3R26Cit peaks is centered on the H3R26Cit summit (red trace). The composite ER signal at H3R26Cit peaks that do not overlap ER peaks (grey trace) is unimodal and centered at the H3R26Cit summit, suggesting that ER is bound to these sites and below our threshold for detection.

**Figure 2:**
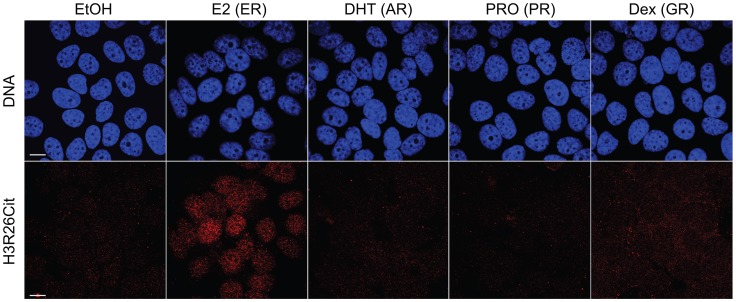
Hormone-induced H3R26Cit is unique to estradiol. H3R26Cit staining is only seen in MCF-7 cells treated with 100 nM Estradiol for 45 minutes. The same concentration and duration of Dihydrotestosterone (DHT), Progesterone (PRO), and Dexamethasone (Dex) did not induce H3R26Cit. Ethanol (EtOH) was used as a control.

We previously demonstrated that PAD2 binds to chromatin and regulates gene expression in MCF-7 cells via histone deimination [13]. Additionally, we have shown that the H3R26Cit modification is important for E2 mediated activation ER-target genes [12]. To directly test whether H3R26 deimination occurred at ER binding sites, we examined the genome-wide relationship of ER binding and H3R26 deimination by performing ChIP-seq of ER and H3R26Cit in MCF-7 cells before and after 40 minutes of E2 treatment. We identified 12,301 ER peaks and 28,495 H3R26Cit peaks and found that both sets of peaks largely overlap with previously identified ER peaks in MCF-7 cells [14], [15] (Figure S1). We found only 9 H3R26Cit peaks prior to E2 stimulation, all of which increase after E2 stimulation, suggesting that E2-induced ER binding is directly or indirectly responsible for 99.97% of the H3R26Cit peaks at any time point. Further inspection reveals that the 9 H3R26Cit peaks prior to E2 treatment are a result of ER binding in the absence of ligand (Figure S2). Ninety-five percent of ER binding sites overlap with H3R26Cit peaks and the intensity of H3R26Cit linearly correlates with ER intensity at ER binding sites (Figure 1A, B). Reciprocally, 47% of H3R26Cit peaks overlap with ER peaks (Figure 1D); and, ER intensity correlates linearly with H3R26Cit intensity at H3R26Cit peaks (Figure 1E). Following E2 treatment, the composite signals for H3R26Cit, H3K4me1, and H3K4me2 are highest at the ER ChIP-seq summit (Figure 1C,F and Figure S3) [3]. In contrast, both AR and GR cause a depletion of modified nucleosomes at the sites of nuclear receptor binding after ligand treatment [3] (Figure S4). These observations support the hypothesis that ER binding occurs at nucleosomal EREs [3]. We next found that the activation of other nuclear receptors, including AR, PR, and GR by their respective ligands does not induce global H3R26 deimination (Figure 2), despite the high expression levels of each receptor in MCF-7 cells (Figure S5). This finding raises the possibility that H3R26 deimination may be unique to ER-activation in MCF-7 cells.

### The H3R26Cit profile is uniquely sharp

**Figure 3:**
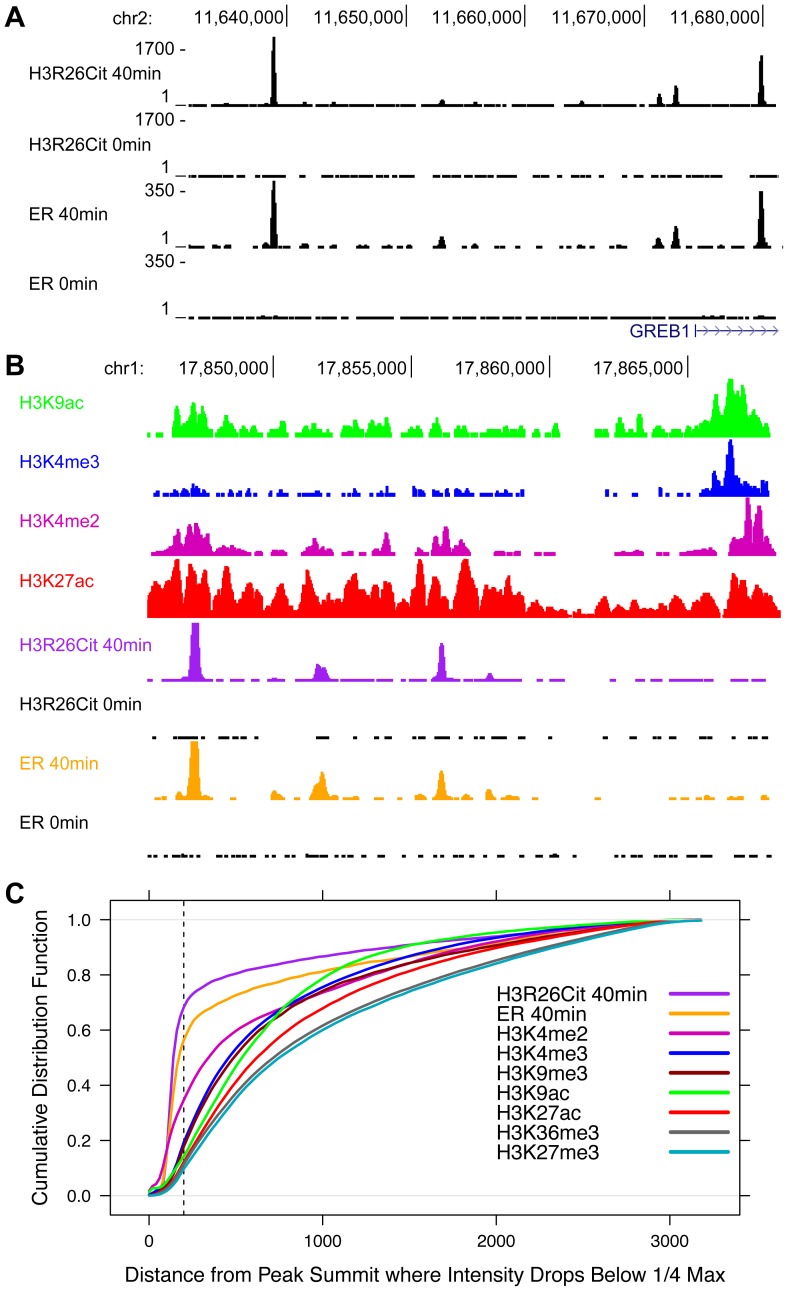
The genomic profile of H3R26Cit is uniquely discrete compared to other histone modifications. A) UCSC genome browser screen shots reveal that ER and H3R26Cit patterns are nearly identical in their position and distribution. B) Other histone modifications, derived from ChIP-seq experiments in MCF-7 cells [3], [16], have broader distributions than H3R26Cit. C) We quantified the discrete nature of all peaks by plotting the cumulative distribution function for the distance from the peak summit whereby the peak reaches 25% the maximal intensity. Note that the ER and H3R26Cit functions are distinct from all other histone modifications.

In order to further highlight the unique peak structure of the H3R26Cit mark, we next compared the H3R26Cit peak structure with the genomic profiles of other histone marks that are associated with active (H3K4me3, H3K4me2, H3K9ac, H3K36me3, and H3K27ac) and repressed (H3K27me3 and H3K9me3) chromatin [16]. A UCSC genome browser screenshot of a representative locus shows that the H3R26Cit profile is unlike any previously characterized histone modification profile in MCF-7 cells, and looks indistinguishable from that of sequence-specific TFs, such as ER (Figure 3A). We quantified the discrete nature of the H3R26Cit peaks and found that 55% of the ER peaks drop to 25% maximum intensity within 200 bp of the summit, while 67% of H3R26Cit peaks are less than 25% maximum by 200 bp. In contrast, the H3K4me2 and H3K4me3 modifications (Figure 3B) have the next most narrow peak structure with only 33% and 18% of the peaks at 25% max within 200 bp of the peak summit, respectively (Figure 3C).

### ER and H3R26Cit have distinct kinetics

**Figure 4:**
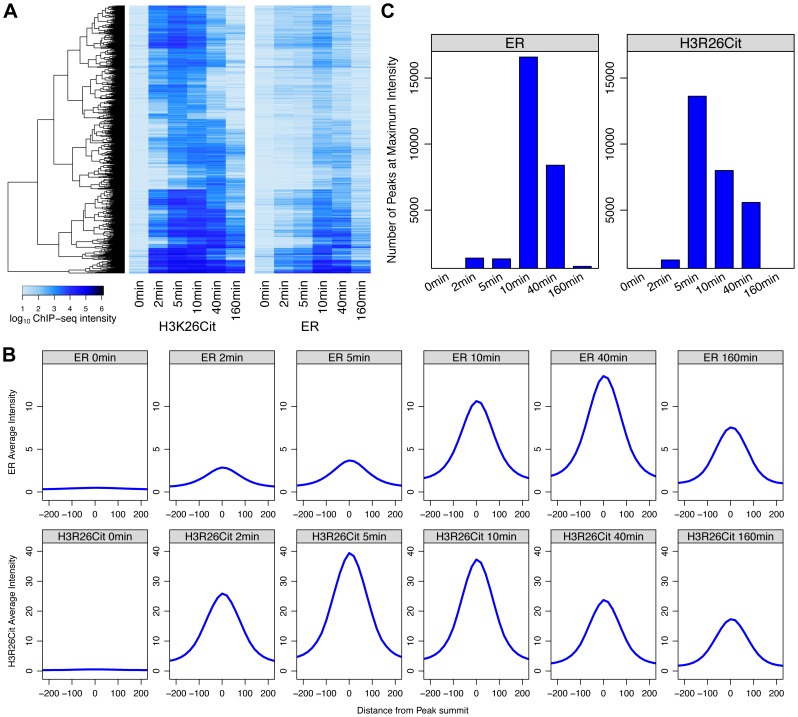
Maximal H3R26Cit peak intensity is early compared to ER and H3R26Cit is required for maximum ER binding. A) Euclidian distance clustering revealed distinct groups of H3R26Cit binding. The ER signals are ordered based on clustering of H3R26Cit kinetic profiles, and the same clusters are distinguishable. B) The average ER signal at H3R26Cit peaks exhibits distinct kinetics from the H3R26Cit signal. C) The H3R26Cit intensity at most H3R26Cit peaks is highest at 5 minutes. However, the ER intensity at most H3R26Cit peaks is highest at 10 minutes.

Next, we compared the kinetics of ER binding and H3R26 deimination following E2 treatment to further elucidate the timing of H3R26 deimination relative to ER binding. The ER intensity and H3R26Cit intensities were compared at each H3R26Cit peak for each time point (Figure S6). The Pearson correlation for each E2-induced time point was between 0.9 and 0.97, indicating that ER binding intensity is quantitatively correlated with H3R26Cit at H3R26Cit peaks. In fact, H3R26Cit peaks that did not overlap with ER peaks correlate linearly as well, suggesting that ER is binding all H3R26Cit sites with the raw ER signal being below our peak calling threshold. These correlations, combined with other analyses (Text S1) lead us to conclude that an H3R26Cit peak is indicative of a site that is bound by ER after E2 stimulation. Therefore, unless noted, we use this set of peaks for the remainder of the analyses (Dataset S1 and Materials and Methods). While we cannot directly compare ChIP signals between H3R26Cit and ER, we are able to compare each antibody's ChIP signal as a function of time. We clustered the H3R26Cit peaks by their H3R26Cit signal; using the same order, we plotted the ER signals on an adjacent heat map (Figure 4A). The H3R26Cit derived clusters are clearly delineated using the kinetic ER data, indicating that the classification of ER binding and H3R26Cit cluster intensities are not appreciably different. However, we found that the average H3R26Cit signal across all peaks is highest at five minutes after E2 treatment and gradually decreases through the remainder of the time course (Figure 4B). In contrast, the average ER signal increases gradually until 40 minutes, and then decreases by 160 minutes after treatment (Figure 4B). We also confirmed that there are more H3R26Cit peaks that are highest at 5 minutes than at any other time point (Figure 4C).

**Figure 5:**
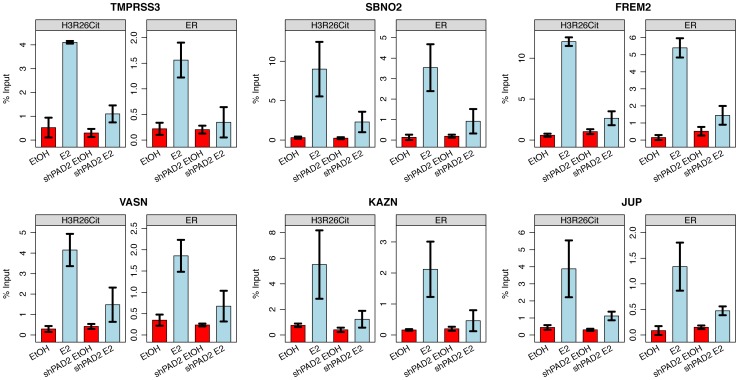
PAD2 depletion abrogates deimination of H3R26 and ER binding. Both H3R26Cit and ER binding is reduced at the TMPRSS3, SBNO2, FREM2, VASN, KAZN, and JUP enhancers. Error bars depict the standard error of the mean of three biological replicates. Similar results are shown for 9 other enhancers (Figure S7).

Over 80% of the ER and 96% of the H3R26Cit signals at the H3R26Cit sites increase at the 2 minute time point, thus making it difficult to definitively determine the temporal sequence of events leading to ER binding. However, the composite profiles and histograms of ER and H3R26Cit signals indicate that H3R26Cit signal reaches maximum intensity at an earlier time point than the ER signal. This finding supports the hypothesis that H3R26Cit plays an important role for the establishment or early maintenance of ER binding. To further test this prediction, we performed ChIP-qPCR at a number of ER binding sites to determine whether stable depletion of PAD2 (the enzyme that deiminates H3R26) resulted in reduced ER binding. Indeed, results show that PAD2 depletion significantly reduces the efficiency of H3R26 deimination and ER binding at these target loci (Figure 5 and Figure S7). Thus, PAD2-mediated H3R26 deimination appears to be required for efficient ER binding to EREs within the context of chromatin.

### H3R26Cit modifies nucleosome structure

To investigate the mechanism by which the H3R26Cit modification facilitates the binding of ER to DNA, we first tested whether hormone treatment promoted gross remodeling events at ER binding sites by examining changes in DNase hypersensitivity following E2 treatment using ENCODE data [17]. To increase the power to detect differential DNase sensitivity we implemented independent pre-filtering of the data, while using the false discovery rate to control for multiple testing [18], [19] (Figure S8 and Figure S9). In contrast to GR, where 39% of GR binding sites show an increase in DNase sensitivity following dexamethasone treatment (Figure S10B) [20], a much smaller fraction of ER binding sites were significantly increased after E2 treatment (Figure S10A). We also find that over 70% of ER-binding sites are DNase hypersensitive prior to E2 treatment. This corroborates a previous study of ER in ECC-1 and T-47D cells, where 72% and 59% of ER binding sites are hypersensitive prior to E2-treatment [21]; this high degree of pre-existing accessibility may partially account for a lower frequency of ER-induced hypersensitive transitions relative to other nuclear receptors. However, the H3R26Cit modification itself does not appear to facilitate ER-binding by promoting the larger scale changes in nucleosomal organization detected as DNase hypersensitive sites. This conclusion is further supported by our analysis of existing datasets which found that E2 treatment does not result in strong depletion of H3-modified nucleosomes at ER-binding sites [3], [16] (Figure S3).

**Figure 6:**
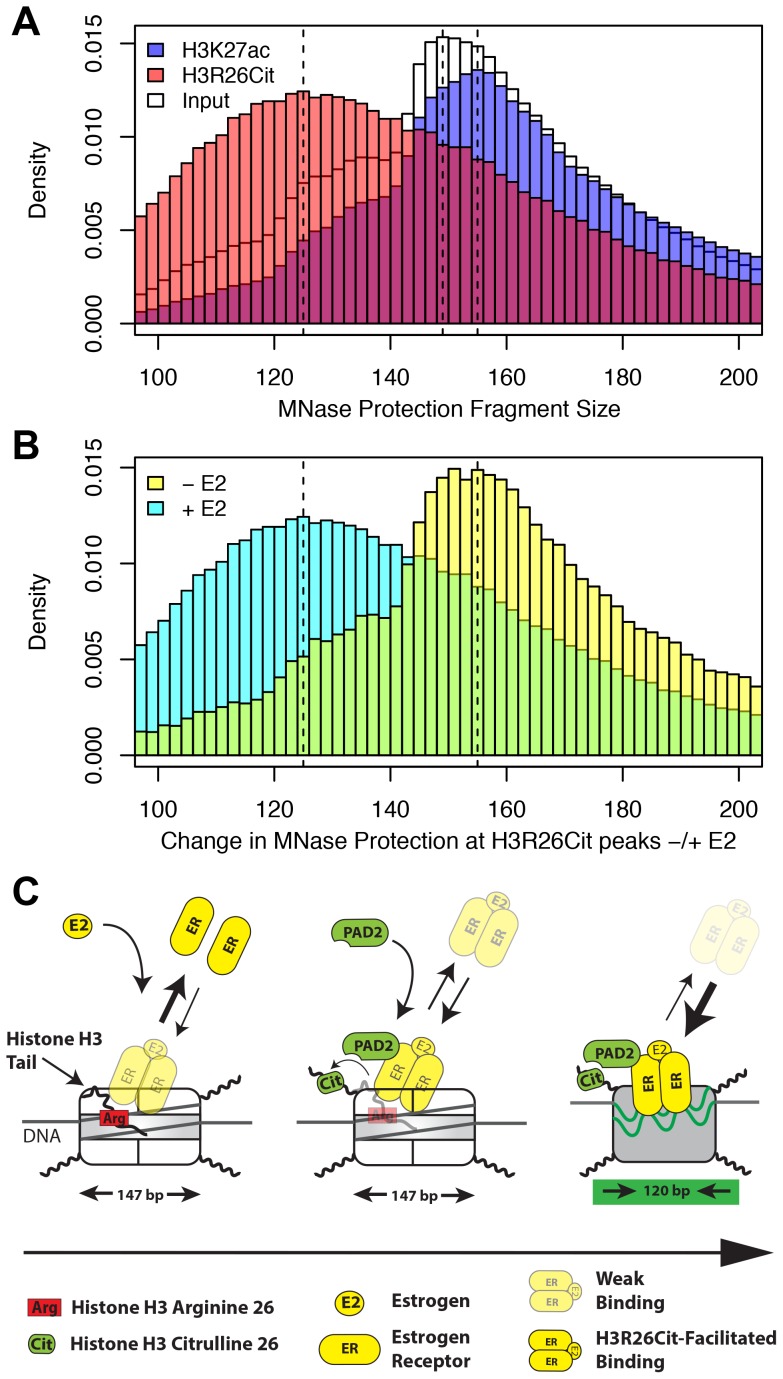
H3R26Cit changes the nucleosome structure to allow for efficient ER binding. A) The MNase protection profiles for input (white), H3K27ac (transparent blue), and H3R26Cit (transparent red) have unique distributions. The dashed lines indicate the modes of the three distributions: 125 bp, 149 bp, and 156 bp. B) The MNase protection profiles at H3R26Cit peaks before and after E2 treatment reveal a shift in MNase protection from 155 bp to 125 bp. C) ER cannot gain access to EREs within nucleosomes containing unmodified histone tails. PAD2 interacts with ER [12] at some frequency as ER gains initial access to EREs. PAD2-mediated H3R26Cit destabilizes the nucleosome structure to facilitate ER binding.

These findings support our principle hypothesis, which is that the H3R26Cit modification enhances the binding of ER to nucleosomal DNA by directly altering nucleosome structure. Previous studies have documented a role for the histone H3 tail in DNA-core particle interaction using MNase digestion [22], [23]. Therefore, we directly tested our hypothesis by performing H3R26Cit ChIP of MNase digested chromatin followed by paired-end sequencing to determine if the H3R26Cit modification altered the MNase-protected region [24]. Results showed that the genomic distribution of protected DNA in the input sample was centered on 149 bp, which is 2 bp larger than would be predicted based on the nucleosome structure [1] (Figure 6A). The MNase protection profile at H3R26Cit peaks prior to E2 exhibited a protection profile with a mode of 155 bp. Importantly, we found that the average protected nucleosome region at H3R26Cit peaks shifts from 155 bp before E2 treatment, to 125 bp after E2-induced deimination (Figure 6B). As a control to test whether other histone modifications are associated with remodeled nucleosomes, we next carried out a control MNase ChIP-seq experiment using an H3K27ac antibody and found that the protected nucleosome region at H3K27ac peaks is 155 bp (Figure 6A) and that this distribution is unchanged upon hyper-acetylation by HDAC inhibition (Figure S11). Note that the protected nucleosome size at ER peaks prior to E2 is also 155 bp because the pre-existing chromatin is largely H3K27-acetylated (Figure S12). These results suggest that PAD2 mediated H3R26 deimination facilitates ER binding by directly altering nucleosomal structure.

Taken together, our findings provide novel insight into the mechanisms by which ER is able to efficiently bind to its DNA element in the context of a chromatin. Our current working model posits that, prior to estrogen treatment, histone H3 arginine 26 (H3R26) interferes with ER-nucleosomal ERE binding by electrostatically interacting with DNA at the ERE. Following E2, ER directly or indirectly recruits PAD2 to nucleosomal EREs where PAD2 then deiminates H3R26, thus neutralizing this residue. Following deamination, the H3 tail then no longer occludes this site allowing for more stable ER-nucleosomal ERE binding (Figure 6C).

### PAD2 and H3R26Cit levels correlate with ER expression in breast tumors and PAD2 expression correlates with survival in patients with Luminal A breast cancer

**Figure 7:**
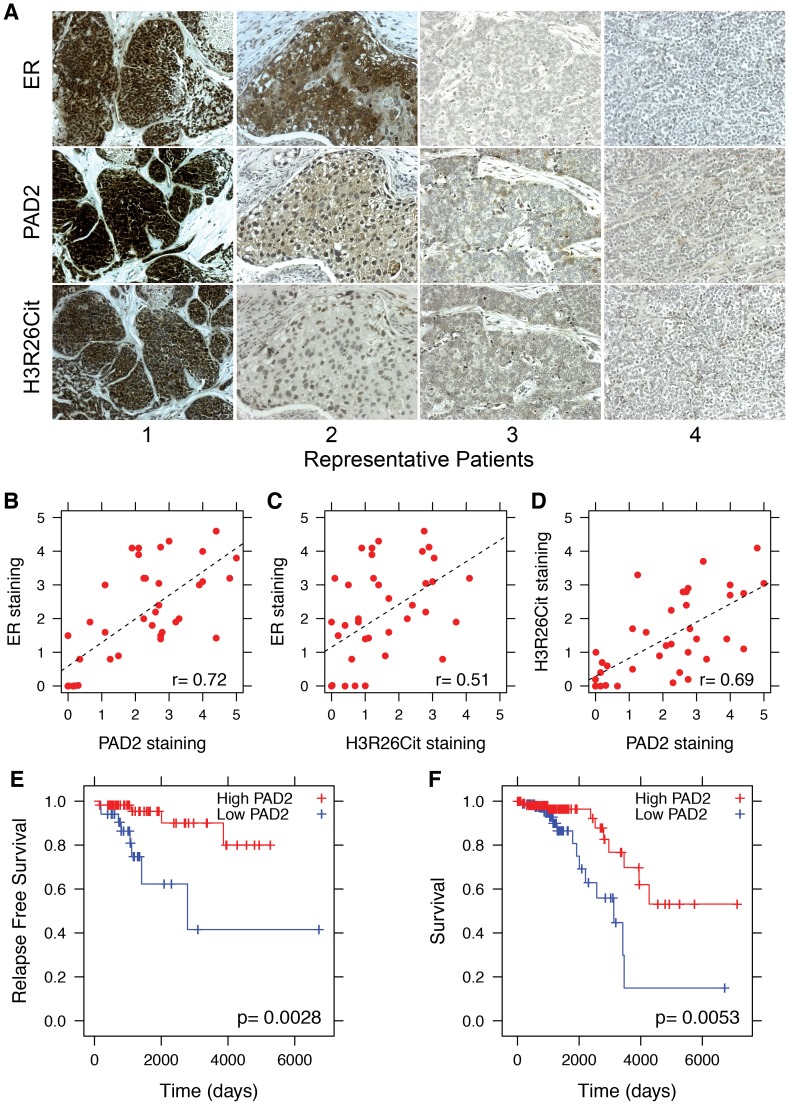
PAD2 expression correlates with breast cancer survival. A) Representative sections from patients who stained positive (1 and 2) and negative (3 and 4) for ER, PAD2 and H3R26Cit. Staining patterns indicate that PAD2 and H3R26Cit is correlated with ER expression; the Pearson coefficient is indicated in lower right of each panel. Staining for B) ER and PAD2 (r = 0.72), C) ER and H3R26Cit (r = 0.51), and D) PAD2 and H3R26Cit (r = 0.69) are significantly correlated. E) Relapse-free survival of Luminal A breast cancer patients with high PAD2 (n = 58) versus low PAD2 (n = 34). F) Overall survival of Luminal A breast cancer patients with high PAD2 expression (n = 112) versus low PAD2 expression (n = 89).

Given that the striking correlation between E2-induced ER binding and H3R26 deimination in MCF-7 cells (a model for ER+ breast cancer), we decided to test whether PAD2 expression and H3R26 deimination may correlate with ER expression in breast tumors. We quantified the coincidence of ER, PAD2, and H3R26Cit staining in serial tumor sections from 21 breast cancer patients. Representative images (Figure 7A) highlight our observation that tumor sections with strong ER signal frequently stain positive for PAD2 and H3R26Cit (e.g. Patients 1 and 2). In contrast, tumors that stain negative for ER rarely stain for PAD2 and H3R26Cit (e.g. Patients 3 and 4). We quantified the staining levels of each section and found that the degree of ER, H3R26Cit, and PAD2 staining between samples is highly correlated (Figure 7B–D). To test whether the observed correlations may have clinical significance, we examined the relationship between PAD2 expression and cancer relapse or overall survival in Luminal A subtype breast cancer patients [25], [26]. We found that PAD2 expression is significantly associated with relapse free survival time [27] (p-value = 0.0028) and overall survival [28] (p-value = 0.0053) (Figure 7E,F). This association is also found by an independent, provisional measure of RNA expression from The Cancer Genome Atlas (Figure S13). These data suggest that PAD2, and possibly the H3R26Cit modification, can be useful as predictive biomarkers to further stratify Luminal A breast cancer patients in terms of their risk of recurrence and overall survival.

## Discussion

Understanding the mechanisms by which transcription factors (TFs) bind to DNA is central to understanding how transcriptional regulatory networks function. We previously showed that ER target gene activation is facilitated by PAD2-catalyzed histone H3R26 deimination. Herein, we provide insight into how H3R26Cit facilitates the ER-DNA interaction, thus providing a mechanistic explanation for the role of PAD2 in E2-induced ER gene activation.

Outcomes from seminal biochemical studies suggested TF access to nucleosomal DNA was hindered by the histone H3 tail, which protrudes from the core histone octamer and overlays nucleosomal DNA [22]. Additionally, these and other studies indicated that neutralization of histone H3 lysine residues via histone acetyltransferase (HAT)-mediated acetylation weakens H3 tail-DNA interactions, thus stabilizing TF-nucleosomal DNA binding. Interestingly, subsequent studies found that deletion of the first 20 amino acids of the H3 tail has no effect on nucleosome stability [29]. However, when more C-terminal regions of the H3 tail were deleted, starting at R26, defects in the wrapping of nucleosomal DNA were observed [29]. Importantly, however, in vitro studies have also found that histone tail hyperacetylation only partially reduces the affinity of histone tails for DNA, likely because the remaining arginine residues are unaffected by acetylation [23], [30]. Outcomes from our study suggest that histone arginine deimination represents a mechanism by which histone H3 arginine residues can be neutralized. Therefore, we suggest that both PAD-mediated histone deimination and HAT-mediated histone H3 tail acetylation are independent and complimentary mechanisms that facilitate TF-nucleosomal DNA binding.

Interestingly, a recent study found that global chromatin decondensation occurs as a result of H1 citrullination by PAD4 upon reprogramming and in the pluripotent cell state [31]. This finding is in line with our previous work showing that PAD4-mediated histone deimination affects nucleosome compaction [32]. However, our results indicate that H3R26 deimination does not result in changes in higher order chromatin structure (Figure S10A, C and Text S1). Instead the core nucleosome particle protection size shifts from 149 bp to 125 bp upon H3R26 deimination. Together, these results support the hypothesis that PAD4 mediated histone deimination mediates nucleosome compaction, while PAD2 mediated histone deimination affects nucleosome structure by modulating the wrapping of DNA around the histone octamer. Similar to that found for other histone modifications, our findings highlight the diverse roles that histone deimination can have upon chromatin structure.

In addition to our more basic findings, this study also demonstrates that PAD2 and histone H3R26 deimination are positively correlated with ER staining in breast tumor sections. We also found that high PAD2 expression is positively correlated with increased relapse-free survival and overall survival in patients with Luminal A breast cancer. Interestingly, a more detailed analysis of the IHC scoring finds that PAD2 and H3R26Cit staining levels are variable in the ER positive sections, with some of the tumors staining strongly for PAD2 and H3R26Cit (e.g. Figure 7A, Patient 1) compared to other tumors (e.g. Figure 7A, Patient 2) stained more weakly for PAD2 and H3R26Cit. Given our finding linking high PAD2 expression with increased survival in ER+ patients, these results suggest that PAD2 and H3R26Cit could help to further stratify ER+ tumors into clinically relevant subsets with respect to outcome.

In sum, our results indicate that the H3R26Cit modification is unlike any previously described histone modification in that the mark is virtually absent from chromatin prior to ligand stimulation and then is observed at ER binding sites across the ER cistrome with over 95% concordance within minutes of estrogen treatment. Additionally, we demonstrate that PAD2-mediated H3R26 deimination appears to facilitate ER-DNA binding by altering the fine structure of the nucleosome. Lastly, our clinical data and analyses indicate that, following further validation, PAD2 and the H3R26Cit modification could be developed into prognostic tools for stratifying ER+ breast cancer patients with respect to survival and treatment.

## Materials and Methods

### Cell culture

MCF-7 cells were maintained in DMEM supplemented with 10% calf serum. The stable PAD2-depleted MCF-7 cell line was described previously [12] and maintained in the medium containing 1 µg/ml puromycin (Sigma). Before hormones treatment, the cells were cultured for 3 days in DMEM phenol red-free medium supplemented with 10% charcoal-dextran-treated calf serum.

### Chromatin Immunoprecipitation (ChIP) and ChIP-seq

ChIP experiments were performed as described previously [12], [33]. Estrogen-starved MCF-7 cells were subjected to E2 treatment at 100 nM for 0, 2, 5, 10, 40 and 160 min, and followed by crosslinking with a final concentration of 2% paraformaldhyde for one minute at 37°C. Crosslinking was quenched in 125 mM glycine on ice for 5 min. Cell lysates were sonicated under conditions yielding fragments ranging from 100 bp to 200 bp. The material was clarified by centrifugation, diluted 10-fold in dilution buffer, and pre-cleared with protein A-agarose beads. The pre-cleared, chromatin-containing supernatant was used in immunoprecipitation reactions with antibodies against H3Cit26 (Abcam, ab19847, lot 135757), ERα (Santa Cruz, sc-542), non-specific rabbit IgG (Upstate 12-370) or without antibody as a control. ChIP-Western analysis confirmed that the H3R26Cit antibody does not recognize ER (Figure S14 and Text S1). The Illumina library preparation was as previously described [34]. Samples were submitted to the Cornell DNA Sequencing and Genotyping Lab and run on the Illumina Genome Analyzer II. Replicates were found to be concordant, with Pearson correlation coefficients all greater than 0.9, for all E2-stimuated data sets where replicates were performed (Figure S15).

### Micrococcal Nuclease (MNase) ChIP-seq

Estrogen-starved MCF-7 cells were treated with ethanol or 100 nM E2 for 10 min. Mononucleosomes were prepared as described previously [12]. The crude chromatin was solubilized with a concentration of MNase (NEB M0247) that produced ∼80% mononucleosomes. The mononucleosomes were then immunoprecipitated using anti-H3Cit26 and anti-H3K27ac (Abcam ab4729) antibodies. Advanced Technology Center (Rockville, MD, USA) performed the Illumina library preparation and the paired-end sequencing.

### qPCR

The primer sequences used for ChIP-qPCR were summarized in Table S1.

### ER and H3R26Cit peaks

Raw sequence reads were aligned to the hg19 genome using bowtie [35]. Replicate concordance was confirmed and replicate files were merged to call peaks using MACS [36] and a mock IP using IgG as a background data set. Broad regions of enrichment were further subdivided using the subpeaks argument in MACS [36]. Subpeaks files from all time points were combined for each antibody. Redundant peaks (those found in more than one time point) were filtered out if a 100 bp window centered on the subpeak summit was within 30 bp of the adjacent subpeak—the subpeak with the most tag counts a the summit position was retained. Raw tag count intensity for each peak coordinate was normalized for each time point and peaks with at least one time point with greater intensity than an independent no antibody ChIP were retained (Dataset S1). We generated composite profile plots by taking the average intensity for a given factor in 20 base pairs steps centered on the peak summits.

### De novo motif analysis

We used MEME with default parameters and 100 bp of sequence information surrounding the peak summit to identify motifs de novo [37]. Despite the fact that many H3R26Cit peaks do not overlap with any ER peaks, the canonical ERE is the most significant motif found de novo at these H3R26Cit peak summits (Figure S16).

### Differential DNase sensitivity

DNase data was downloaded from ENCODE (wgEncodeUwDnaseMCF-7Estctrl0hAlnRep*.bam and wgEncodeUwDnaseMCF-7Est100nm1hAlnRep*.bam).

We used the program DEseq [19] to statistically determine differentially DNase sensitivity at all H3R26Cit peaks using the raw number of reads at each peak before and after estrogen treatment. We applied an independent gene filter to the peaks in order to increase the power to detect differences and still control for multiple testing (Figure S8). The joint distribution of p-values and read count total remains unchanged after filtering, indicating that unadjusted p-value and total reads counts for each peak are independent variables (Figure S9).

### MNase ChIP-seq analysis

Paired-end reads were mapped to the hg19 genome using bowtie2 [35]. Concordant paired-end reads that result in fragment lengths >1 and <500 were considered in the analysis and shown in Figure 6.

### Confocal microscopy

MCF-7 Cells grown on slides were subjected to hormones treatment at 100 nM for 45 min, including Estradiol (E2), Dihydrotestosterone (DHT), Progesterone (PRO), and Dexamethasone (Dex). Ethanol (EtOH) was used as a control. Confocal microscopy experiments were described previously [11]. In short, after hormones treatment, cells were fixed with a paraformaldehyde fixing solution (1×PBS, 0.1% Triton X- 100, 0.2% NP-40, and 3.7% paraformaldehyde) for 10 min at room temperature. After 3 washes (10 min each) with PBST (1×PBS with 0.2% Triton X-100), cells were blocked with 5% BSA in PBST for at least 1 hr at room temperature. The cells were stained overnight at 4°C with anti-H3Cit26 (Abcam, ab19847) antibody diluted in PBST (1∶100). DNA was stained with DAPI (4,6- diamidino-2-phenylindole) before mounting. Images were collected with LSM 510 laser scanning confocal microscope (Carl Zeiss).

### Kaplan-Meier survival analysis

PAD2 expression levels were measured by provisional RNA-seq (Figure S13) data from The Cancer Genome Atlas (TCGA) [25], which was curated by UCSC [26], and published TCGA microarray data (Figure 7A,B) [25]. P-values were automatically selected for the best expression threshold cut-off and calculated using the log-rank test statistic. In Figure 7E and 7F, patient samples were split into two groups according to level of PADI2 expression by above and below the 45% quantiles. In Figure S13A,B patient samples were split into two groups according to level of PADI2 expression by above and below the 65% and 60% quantiles, respectively.

### Immunohistochemistry

Four sections from each breast cancer patient were deparaffinized, unmasked, blocked and treated with either rabbit anti-PADI 2 (Proteintech, 12110-1-ap), rabbit anti-ER-α (Santa Cruz, sc-542), rabbit anti-Histone 3 Citrulline 26 (AbCam, ab19847) or non-specific rabbit IgG (Millipore, S-20) using standard methods and developed using the Vectastain Peroxidase Rabbit IgG and Vector DAB substrate kits. Sections stained for ER were examined using a Zeiss AxioObserver microscope and two tumor cell areas on each section which stained positive were chosen and imaged at 5, 10 and 20× using a Zeiss Axiophot color camera. These same two areas were imaged on the corresponding sections stained for PAD2, H3R26Cit and the IgG negative control. For patients who were negative for ER in all areas of the section, two areas containing tumor cells were chosen for imaging and corresponding images from the remaining slides were captured as above.

In each image the brown DAB staining was scored from 0 to 5, with 0 being no staining and 5 being heavy staining in both nucleus and cytoplasm. The percent of tumor cells in the 10× image at each score level was established and multiplying the stain scores by their corresponding fraction and adding the products together determined a final score for that section area. The following comparisons were made using all of the final scores from both areas of all sections, ER:PADI2, ER:H3R26Cit, PADI2: H3R26Cit.

### Accession numbers

H3R26Cit and H3K27ac ChIP-seq data was deposited into GEO with the accession number GSE58177. ER ChIP-seq data was previously published [38] with accession number GSE54855.

## Supporting Information

Dataset S1 The set of filtered peaks (see Materials and Methods) used in this study.
(TXT)

Figure S1 Previous reports identified 10196 and 14498 peaks [14], [15] compared to our 12301 ER peaks. 66% of our ER and 45% of our H3R26Cit peak summits overlap with previously identified ER peaks. Despite the inability to detect ER at the H3R26Cit peaks in our ChIP-seq, many H3R26Cit peaks overlap with ER peaks found from other studies.
(EPS)

Figure S2 (A) The average ER-ChIP-seq intensity at the 9 pre-E2 H3R26Cit peaks is centered at the H3R26Cit summit. (B) A strong consensus ERE is identified de novo at the pre-E2 H3R26Cit peaks.
(EPS)

Figure S3 The average H3K4me1 [16] and H3K4me2 [3] signal at ER peaks is only modestly reduced after ER binding and does not show a bimodal distribution after E2.
(EPS)

Figure S4 The average modified H3 signal at GR peaks reduces intensity after GR binding and shows a bimodal distribution.
(EPS)

Figure S5 RNA-seq analysis from ENCODE reveals that Estrogen Receptor (A), Glucocorticoid Receptor (B), Progesterone Receptor (C), and Androgen Receptor (D) are all highly expressed in MCF-7 cells.
(EPS)

Figure S6 The ER raw intensity is strongly correlated with H3R26Cit intensity at H3R26Cit peaks at all E2-induced time points: 0 min (A), 2 min (B), 5 min (C), 10 min (D), 40 min (E), and 160 min (F).
(TIF)

Figure S7 PAD2 depletion abrogates deimination of H3R26, resulting in compromised ER binding.
(EPS)

Figure S8 A) The total read counts between all conditions for a given region inversely correlates with the unadjusted p-value test statistic. We found that the bottom 50% of regions, in terms of total tag counts, do not have an unadjusted p-value below 0.002, and thus has a small chance of showing statistical significance after correcting for multiple testing at over 28000 regions. So we used this 50% tag count threshold as a starting point for pre-filtering the data. B) We assessed the power to detect significant changes by pre-filtering between 30% and 70% of the data. We found that we gradually increase statistical power as we remove 30%, 40%, and 50% of the data. We concluded the 50% threshold was reasonable, as we start to over-filter as we go to 60% (the 60% line dips below the 50% line). This is a result of discarding true positives in addition to false positives at higher thresholds. C) This panel confirms that 50% is a reasonable filter statistic because the ability to detect changes in DNase begins to level out at 50% for a range of false discovery rates (FDR). D) We report a false discovery rate (FDR) of 0.15 within the text, because over half of the newly added regions as we change the FDR from 0.05 to 0.10 and to 0.15 are estimated to be true positives, but less than half of the newly added regions are estimated to be true positives as we increase the FDR beyond 0.15.
(EPS)

Figure S9 We confirmed that the unadjusted p-value and total reads counts for a region are independent by showing that the joint distribution of p-values and read count total does not change after filtering. Panel A illustrates the distribution of unadjusted p-values for all regions. Panel B shows the same result, but the fraction of regions that are filtered by our independent filter statistic are colored white. Panel C shows the distribution of the filtered regions, which is successful at removing the background set of regions without affecting the overall distribution.
(EPS)

Figure S10 A) Only 122 (<0.5%) H3R26Cit peaks significantly change their DNase signal after E2 treatment. B) In contrast 39% of GR peaks show significant changes in DNase signal [20]. Genomic changes in DNase hotspots are also modest after E2 treatment (C) compared to dexamethasone treatment (D).
(TIF)

Figure S11 TSA treatment does not alter the MNase protection profiles for H3K27ac; the modes of the distributions are 156 bp prior to TSA and 155 bp after TSA treatment.
(EPS)

Figure S12 Eighty-nine percent of ER peaks that are also enriched for H3R26Cit and have more than 20 sequence tags in the peak summit position are enriched for H3K27ac prior to E2 treatment.
(EPS)

Figure S13 A) Relapse-free survival of Luminal A breast cancer patients with high PAD2 (n = 40) versus low PAD2 (n = 61). B) Overall survival of Luminal A breast cancer patients with high PAD2 expression (n = 90) versus low PAD2 expression (n = 132).
(EPS)

Figure S14 Immunoprecipitation and subsequent Western with anti-ER antibody and an anti-light chain IgG secondary antibody (Western) detect ER. Note that ER and the heavy chain IgG are similar sizes, so an anti-light chain is necessary when probing the Western blot. In contrast, the H3R26Cit antibody does not cross-react with immunoprecipitated ER.
(TIF)

Figure S15 ER and H3R26Cit ChIP-seq replicates are highly concordant and strongly correlated for all replicate data: ER 0 min (A), ER 10 min (B), ER 40 min (C), H3R26Cit 0 min (D), H3R26Cit 10 min (E), H3R26Cit 40 min (F), MNase H3R26Cit 5 min (G), and MNase H3R26Cit 10 min (H).
(TIF)

Figure S16 The canonical ERE is the most significant motif found de novo at H3R26Cit peaks where no ER peak has ever been detected by ChIP-seq in all three studies.
(EPS)

Table S1 Primers for qPCR used in this study.
(XLSX)

Text S1 Complementary and additional analyses indicate that H3R26Cit specifically marks ER binding sites, ER binding induces a concomitant increase in DNase sensitivity at only a small fraction of ER binding sites, and that the H3R26Cit antibody does not cross-react with ER.
(DOCX)
