# Supplementary figures and images for: Targeted H3R26 Deimination Specifically Facilitates Estrogen Receptor Binding by Modifying Nucleosome Structure
Source: PLoS Genet. 2014 Sep 11;10(9):e1004613. doi: 10.1371/journal.pgen.1004613 (PMC4161307; doi:10.1371/journal.pgen.1004613)

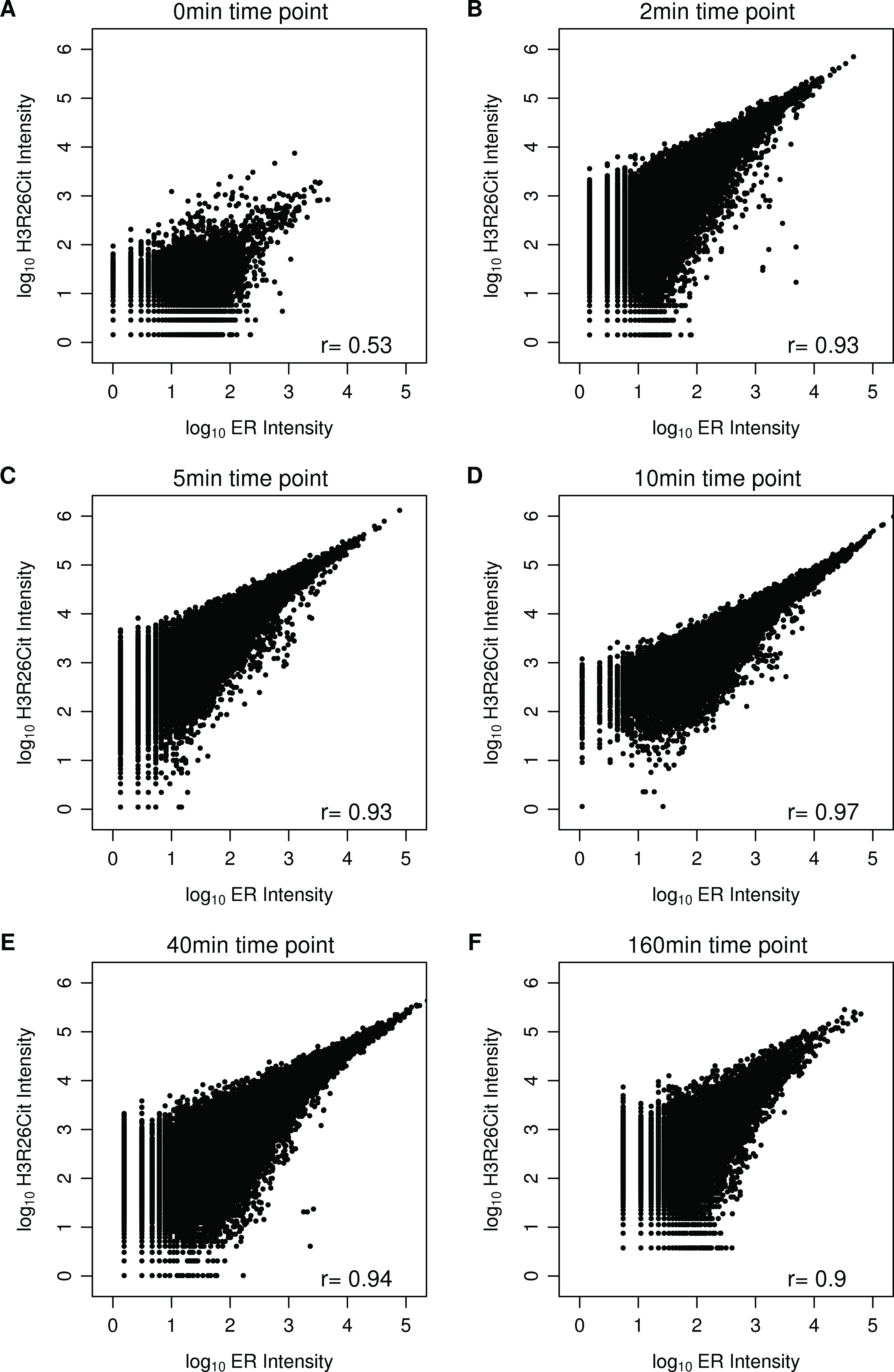

Supplement: Figure S6 — The ER raw intensity is strongly correlated with H3R26Cit intensity at H3R26Cit peaks at all E2-induced time points: 0 min (A), 2 min (B), 5 min (C), 10 min (D), 40 min (E), and 160 min (F). (TIF) [file pgen.1004613.s007.tif]

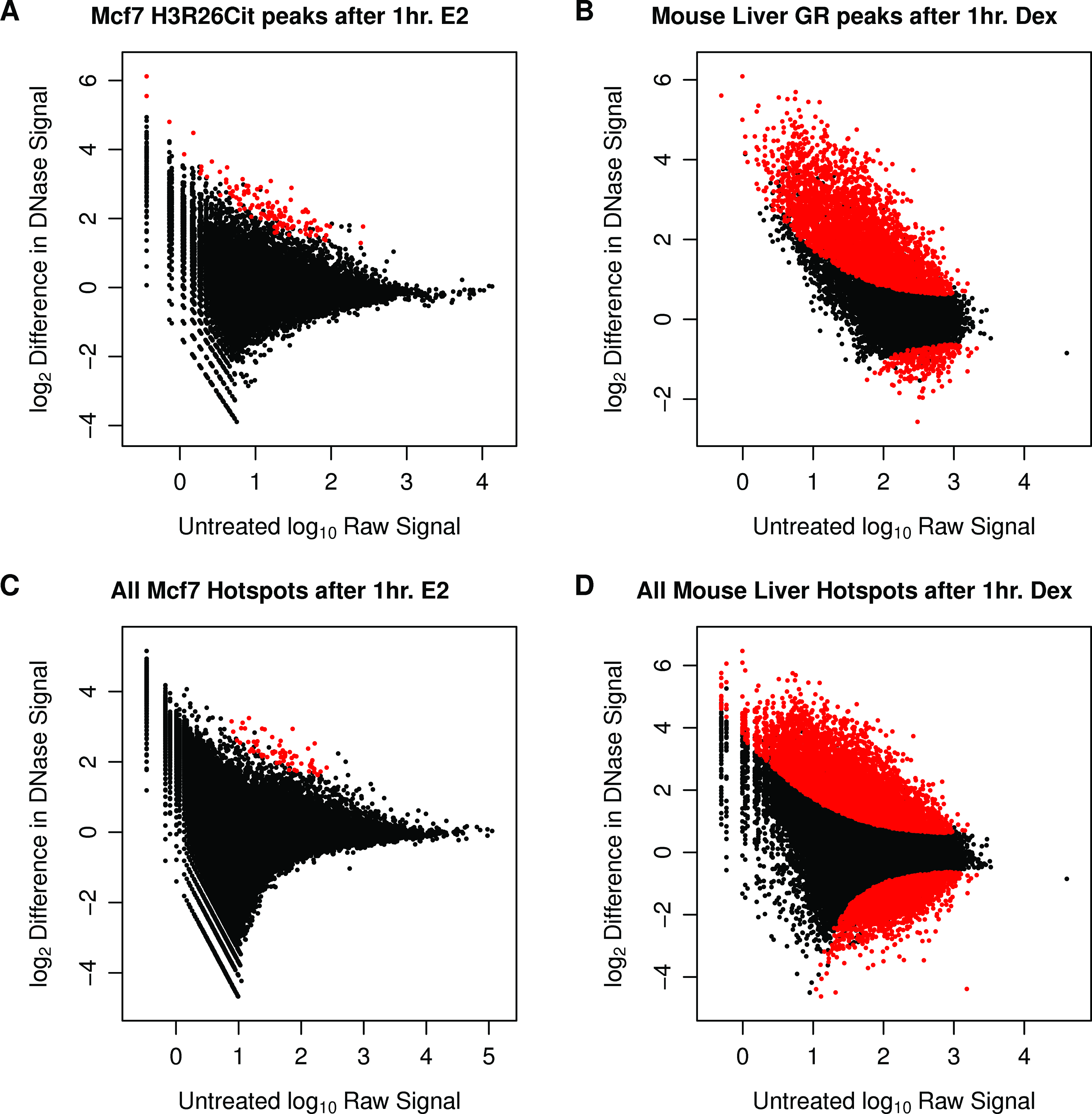

Supplement: Figure S10 — A) Only 122 (<0.5%) H3R26Cit peaks significantly change their DNase signal after E2 treatment. B) In contrast 39% of GR peaks show significant changes in DNase signal [20]. Genomic changes in DNase hotspots are also modest after E2 treatment (C) compared to dexamethasone treatment (D). (TIF) [file pgen.1004613.s011.tif]

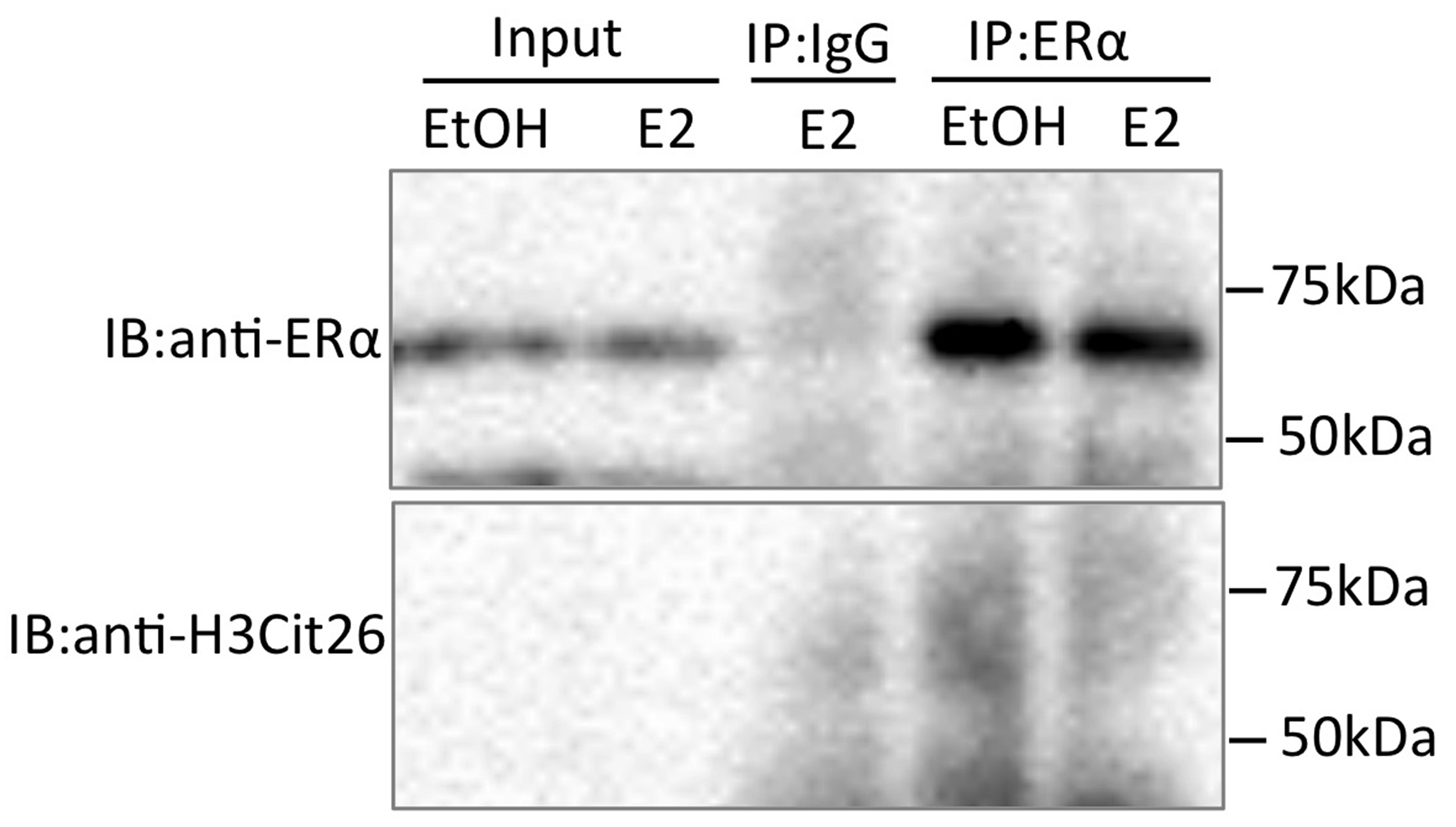

Supplement: Figure S14 — Immunoprecipitation and subsequent Western with anti-ER antibody and an anti-light chain IgG secondary antibody (Western) detect ER. Note that ER and the heavy chain IgG are similar sizes, so an anti-light chain is necessary when probing the Western blot. In contrast, the H3R26Cit antibody does not cross-react with immunoprecipitated ER. (TIF) [file pgen.1004613.s015.tif]

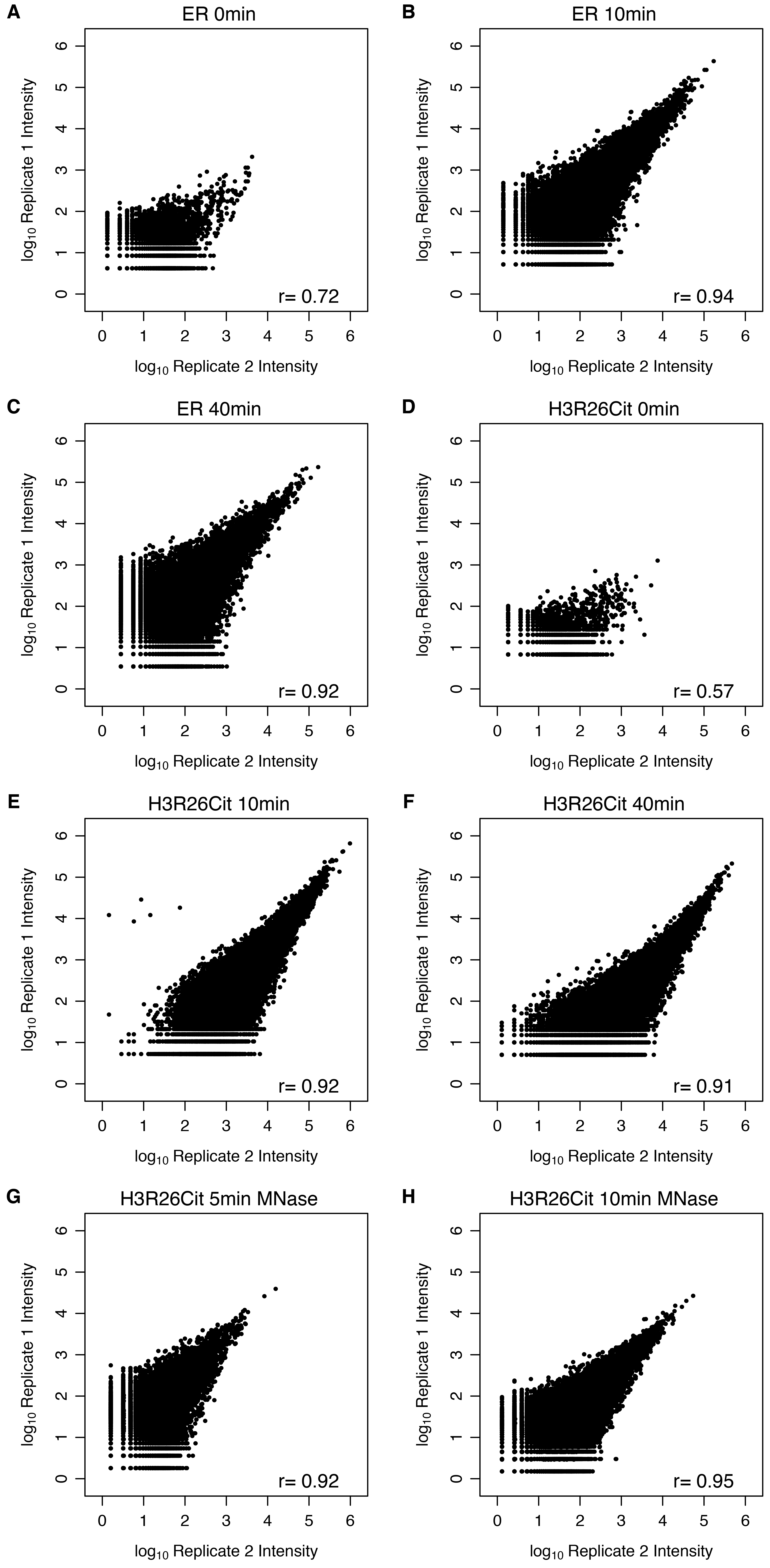

Supplement: Figure S15 — ER and H3R26Cit ChIP-seq replicates are highly concordant and strongly correlated for all replicate data: ER 0 min (A), ER 10 min (B), ER 40 min (C), H3R26Cit 0 min (D), H3R26Cit 10 min (E), H3R26Cit 40 min (F), MNase H3R26Cit 5 min (G), and MNase H3R26Cit 10 min (H). (TIF) [file pgen.1004613.s016.tif]
