# Supplementary material for: Targeted H3R26 Deimination Specifically Facilitates Estrogen Receptor Binding by Modifying Nucleosome Structure
Source: PLoS Genet. 2014 Sep 11;10(9):e1004613. doi: 10.1371/journal.pgen.1004613 (PMC4161307; doi:10.1371/journal.pgen.1004613)
Supplement: Text S1 — Complementary and additional analyses indicate that H3R26Cit specifically marks ER binding sites, ER binding induces a concomitant increase in DNase sensitivity at only a small fraction of ER binding sites, and that the H3R26Cit antibody does not cross-react with ER. (DOCX) [file pgen.1004613.s019.docx]

**Supporting Text**

**H3R26Cit marks sites of ER binding**

In addition the linear correlations of raw ER and H3R26Cit signal at all H3R26Cit peaks, we performed other analyses to come to the conclusion that H3R26Cit exclusively marks ER binding sites. For instance, the composite ER signal at H3R26Cit peaks that do not overlap ER peaks is unimodal and centered at the H3R26Cit summit, suggesting that ER is bound to these sites and below our threshold for detection (Figure 1F). We next checked to see if H3R26Cit peaks that do not overlap with our ER ChIP-seq peaks do overlap with previously identified ER binding sites from other studies [14, 15] (Figure S1). Indeed we find that H3R26Cit peaks overlap extensively with previous ER ChIP-seq peaks (Figure S1). Furthermore, the Estrogen Response DNA Element (ERE) was the most significant motif found *de novo* at H3R26Cit peaks that did not overlap with any ER ChIP-seq from this study or previous studies [14,15] (Figure S15).

**Post-E2 DNase changes are limited to a subset of ER-binding sites**

The majority (over 70%) of ER binding sites are DNase hypersensitive in the MCF-7 genome prior to E2-treatment, but few sites change in hypersensitivity after E2 treatment (Figure S10C). This is in contrast to GR in mouse liver cells [20] (Figure S10D), or mouse mammary cells [4], where glucocorticoid receptor (GR) binding sites and hotspots show significant changes in DNase hypersensitivity after treatment with dexamethasone. Chromatin changes at 95% of ER binding sites are associated with H3R26 deimination. Note, that among the significant changes of DNase hypersensitivity that occur at ER binding sites, we find TFF1 (pS2), which is in agreement with previous reports [7]. Our results confirm other studies that show ER-mediated recruitment of the SWI/SNF remodeling complex to this site [8]. Furthermore, ER can act differently in different cell types. For instance, in ECC-1 and T-47D cells ER does cause significant changes in hypersensitivity at 5% and 21% of ER binding sites [21]. The functional relationship between deimination, ATP-dependent remodelers, and histone chaperones remains to be determined.

**The H3R26Cit antibody does not cross-react with ER**

We have previously published data that supports the specificity of the H3R26Cit antibody. Previously we had treated cells +/- E2, and we observed a band at 17 kDa using the H3R26Cit antibody [12]. This is consistent with the immunoflorescence data that shows E2-dependent H3R26Cit staining, which is absent without E2 (Figure 1). In our previous publication we also treated cell-derived histones with recombinant PAD2 and then probed with H3Cit26 antibody, we found a sharp and strong band at 17 kDa [12]. This band was absent in the histones without PAD2 treatment, indicating that PAD2 citrullinates histone H3; subsequent mass spectrometry analysis of this band showed that H3R26 was citrullinated. Additionally, the antibody against ER can identify immunoprecipitated ER, but the H3R26Cit antibody does not recognize ER (Figure S16).
